# Monitoring Resistance Training in Real Time with Wearable Technology: Current Applications and Future Directions

**DOI:** 10.3390/bioengineering10091085

**Published:** 2023-09-14

**Authors:** Toon T. de Beukelaar, Dante Mantini

**Affiliations:** Movement Control and Neuroplasticity Research Group, Department of Movement Sciences, KU Leuven, 3001 Leuven, Belgium; toon.debeukelaar@kuleuven.be

**Keywords:** telemonitoring, sport, performance, training, movement, muscle activity, cardiac activity, exercise physiology

## Abstract

Resistance training is an exercise modality that involves using weights or resistance to strengthen and tone muscles. It has become popular in recent years, with numerous people including it in their fitness routines to ameliorate their strength, muscle mass, and overall health. Still, resistance training can be complex, requiring careful planning and execution to avoid injury and achieve satisfactory results. Wearable technology has emerged as a promising tool for resistance training, as it allows monitoring and adjusting training programs in real time. Several wearable devices are currently available, such as smart watches, fitness trackers, and other sensors that can yield detailed physiological and biomechanical information. In resistance training research, this information can be used to assess the effectiveness of training programs and identify areas for improvement. Wearable technology has the potential to revolutionize resistance training research, providing new insights and opportunities for developing optimized training programs. This review examines the types of wearables commonly used in resistance training research, their applications in monitoring and optimizing training programs, and the potential limitations and challenges associated with their use. Finally, it discusses future research directions, including the development of advanced wearable technologies and the integration of artificial intelligence in resistance training research.

## 1. Introduction

Resistance training is a popular exercise modality that involves external resistance to improve muscular strength, endurance, and hypertrophy [1]. When engaging in resistance training, body muscles are contracted forcefully against an external resistance to cause muscle fibers to have microscopic tearing. The muscle tears heal during periods of rest which results in a larger and stronger muscle (i.e., hypertrophy). Additionally, resistance training can help to improve muscle endurance, allowing individuals to perform physical activities for longer periods of time without augmented fatigue.

Resistance training used to be considered only for athletes involved in strength-dependent sports like weightlifting and bodybuilding. Unmistakably, these athletes clearly need high levels of strength and muscle to perform in their chosen sport. More so, the average person has rarely seen reasons to engage in weight training, and participants in other sports have often felt that lifting weights is detrimental to their athletic performance. However, recent studies have shown that endurance athletes can also benefit from resistance training [2]. More specifically, it improves muscular endurance and efficiency, which helps maintain good form and reduces the risk of injury during long events. Additionally, resistance training can improve running economy, allowing athletes to use less energy to maintain a given pace and perform better during endurance events [1]. Outside of sports, resistance training has gained more interest in relation to aging. Studies have linked age-related muscle loss to other health issues such as bone loss, metabolic decline, fat gain, diabetes, and metabolic syndrome [3]. Resistance training has been shown to promote muscle growth, bone density, balance, and co-ordination, thereby reducing the risk of fall accidents and injuries in people of all ages. As a result, resistance training programs are being suggested as a way to improve overall health [2,4].

Each type of resistance training has its own unique benefits and challenges, and the general goal of resistance training research is to identify the combination of exercises and training protocols that can help maximize overall strength and fitness [5]. This can most optimally be achieved by using information on the current health status of the individual, as well as on training goals. Technology advancements in recent years have permitted the use of wearables for telemonitoring and remotely gathering data about body movement and physiology during resistance training [6,7]. Wearable sensors used in resistance training include smart watches to measure heart rate, burned calories, and position over time. Smart clothes can track body movements and muscle activation during resistance training workouts. Specialized devices can be attached to the body to measure electrocardiographic activity and muscle oxygenation. Other sensors can be integrated into training equipment to track movements and forces. All wearables are wirelessly synced with a computer or a smartphone, through which data are uploaded to online repositories for long-term storage and offline analyses.

This review aims to summarize the current state of wearable technology in resistance training research and highlight its potential benefits and limits. It will first describe the general principles of resistance training, and, subsequently, discuss wearable devices for measuring physiological and biomechanical parameters during resistance training. It will examine the primary applications of wearables in monitoring and optimizing training programs and will present the limitations and challenges associated with their use. Finally, it will also discuss future research directions, including developing novel advanced wearable technologies and integrating artificial intelligence in resistance training research.

## 2. General Principles of Resistance Training

Resistance training, also known as strength or weight training, is a form of exercise that focuses on improving muscular strength, muscle mass, endurance, and overall physical fitness. It can typically be implemented through (1) free weights, used to perform exercises such as squats, deadlifts, and bench presses; (2) weight machines, designed to target specific muscle groups and provide a controlled range of motion; (3) bodyweight exercises, which use the bodyweight to provide resistance; (4) resistance bands, used to perform a variety of exercises such as bicep curls, shoulder presses, and leg extensions; (5) suspension exercises, using specialized straps to perform bodyweight exercises that target multiple muscle groups simultaneously; and (6) plyometric exercises, involving explosive movements that use bodyweight to build explosive power and strength.

Resistance training offers numerous advantages for individuals of all ages and fitness levels and is an effective way to improve overall health. More specifically, it renders building and strengthening of muscles, stimulates bone tissue production, and improves bone density. It also enhances joint stability and constitutes an effective tool for weight management. Moreover, particular resistance training exercises can provide cardiovascular benefits and regular strength training can have a positive impact on mental health and sleep quality [2,8,9,10].

### 2.1. Progressive Overload

Progressive overload constitutes the foundation of resistance training. It involves gradually increasing the stress placed on the muscles over time to stimulate muscle growth, strength gains, and/or endurance. This can be achieved by mediating different training variables based on the frequency, intensity, type, and time (FITT) principles [11]. Hereby a gradual increase in the number of repetitions or sets performed; a progressive increase in the weight lifted during exercises; an intensified exercise through reduced rest periods, advanced techniques, or more challenging variations; and/or an increased frequency of resistance training sessions generate further muscle growth and adaptation [12].

### 2.2. Specificity

The principle of specificity in resistance training emphasizes the need for tailored training programs aligned with specific goals, movements, and muscles. Adherence to this principle acknowledges that the body adapts to the specific demands imposed upon it. Training methods should be customized based on different objectives. To enhance muscular strength, heavy weights, and lower repetitions are prioritized, whereas improving muscular endurance requires lighter weights and higher repetitions. By aligning training methods with desired outcomes, workout effectiveness will be optimized. Specific exercises target and strengthen specific muscle groups or movement patterns. Training movements that mimic desired activities or sports enhances performance in those areas. To develop specific muscles or muscle groups, exercises focusing on those areas, such as bicep curls and chin-ups for bicep strengthening, can be employed. Selecting exercises that isolate and emphasize specific muscles effectively stimulates their growth and development.

Distinct activities rely on different energy systems. Enhancing performance in a specific sport or activity necessitates considering the energy demands associated with it and training accordingly. Endurance athletes may integrate longer duration, lower intensity exercises to enhance aerobic capacity, while sprinters may focus on high-intensity interval training to develop anaerobic power [13].

### 2.3. Variation

Variation in resistance training encompasses deliberate modifications to exercises, parameters, or training methods within a workout program. It serves as a crucial principle in preventing plateaus, sustaining motivation, and nurturing continuous progress and adaptation. Strategies for incorporating variation in resistance training include exercise diversification, alteration of repetition ranges, utilization of diverse training methods, manipulation of lift tempo, and adjustment of training split and exercise order. By implementing these approaches, individuals engage muscles from different angles, challenge the nervous system, diversify stimuli, and maintain training efficacy [14].

### 2.4. Individualization

Individualization in resistance training involves tailoring the program to address the unique needs, goals, abilities, and limitations of individuals. It begins with a comprehensive assessment of one’s goals, current fitness level, medical history, movement patterns, and specific considerations. This information informs the development of a customized program that aligns with the individual objectives and accommodates any factors affecting the training.

Essential to individualization is the regular monitoring of progress and adjusting the training program accordingly. These updates can be based on feedback via assessments of strength gains (e.g., one-repetition maximum (1RM), which is the maximum weight an individual can lift for a single repetition), body composition changes (e.g., changes in total body fat percentage or total lean mass), performance improvements, or any other relevant metric [14].

### 2.5. Proper Form and Technique

Proper technique is vital in resistance training to ensure safety, optimize effectiveness, and prevent injuries. Key principles include maintaining good posture and alignment, emphasizing controlled movements, performing a warm-up prior to training, practicing proper breathing techniques, avoiding excessive weights that compromise form, and starting with exercises suitable for one’s fitness level and gradually progressing. By adhering to these principles, the benefits of resistance training can be enhanced while minimizing the risk of injuries [14].

### 2.6. Rest and Recovery

Rest and recovery are vital for effective resistance training as they allow the body to repair, adapt, and maximize training benefits. Training stress induces fatigue, a state in which physical and/or mental performance is reduced because of disturbances in biopsychosocial homeostasis. In the specific context of resistance training, this results in small tears in the muscle fibers, which then initiate the repair process during periods of rest. Adequate rest allows for the effective restoration of muscles, fostering their growth and enhancing their strength.

In case the stress and recovery balance are not sufficiently considered, one could evolve from a state of functional to dysfunctional over-reaching, and eventually progress to a state of overtraining. The latter is a chronic imbalance between stress and recovery and this physiological state hampers performance and raises injury risks. Hence, adequate sleep, proper nutrition, active recovery, scheduled rest days, and periodic deloading (i.e., periodization) are crucial practices for effective recovery and injury prevention in resistance training [15,16].

## 3. Wearable Devices for Measuring Physiological Parameters during Training

Wearable devices have become increasingly popular for monitoring and measuring physiological parameters due to their convenience and accessibility. These devices are designed to be worn on the body and typically integrate sensors and technologies that allow for the measurement and tracking of various physiological metrics. Accordingly, wearable devices are classified in several countries as medical devices, for which they need to undergo a conformity assessment. This involves an audit of the manufacturer’s quality system and, depending on the type of device, a review of technical documentation on its safety and performance [17,18].

**Figure 1:**
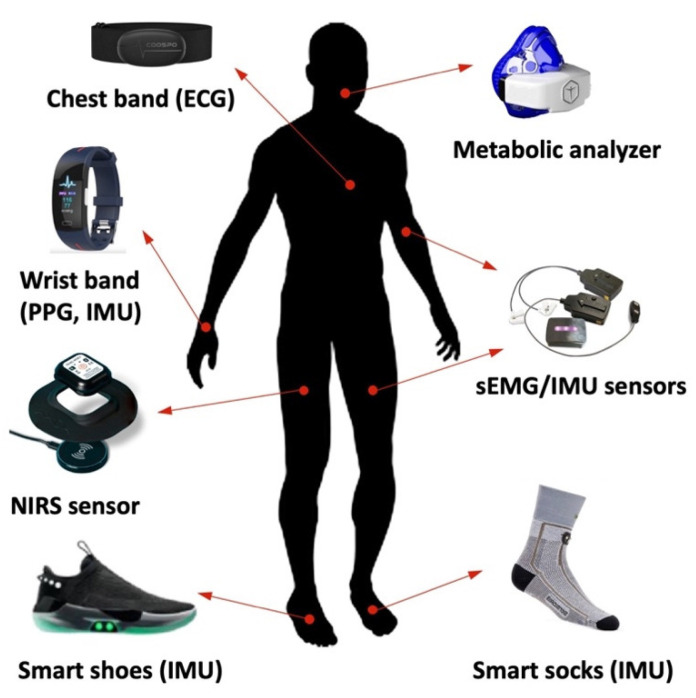
Portable and healthcare devices worn on body parts, which can be used in resistance training research. Specific sensors are integrated into wearable systems (e.g., wrist and chest bands, and mart shoes and socks), whereas other sensors are directly positioned over the body using elastic bands or adhesive material. ECG: electrocardiography; PPG: photoplethysmography; NIRS: near-infrared spectroscopy; sEMG: surface electromyography; IMU: inertial measurement unit.

A specific subset of these devices has shown to be promising for use in resistance training protocols (Figure 1), including wrist and chest bands, smart socks and shoes, and specific sensors to measure tissue oxygenation, cardiac and muscular activity, and movement [19]. The combined use of those sensors can provide valuable information on body physiology, aiming at tracking the individual’s physical condition and optimizing the workout.

### 3.1. Photoplethysmography for Heart Rate Monitoring

**Figure 2:**
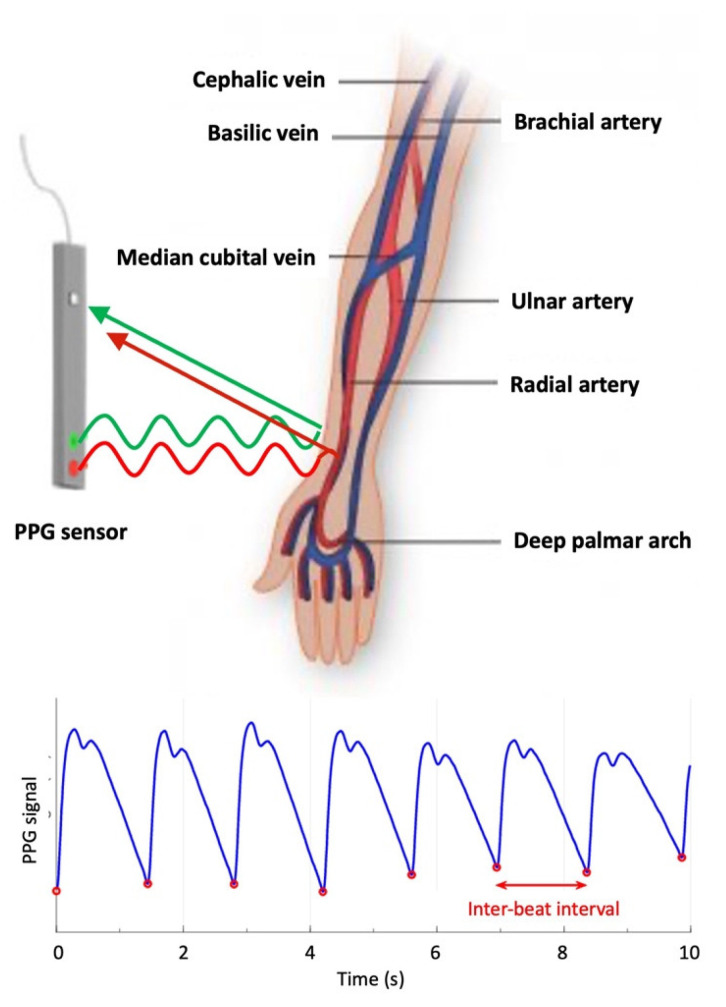
Schematic representation of a PPG detection system, along with a sample PPG signal with red circles indicating individual heartbeats.

Photoplethysmography (PPG) is a non-invasive optical technique that can be used to measure the real-time variation of clinically relevant physiologic parameters and extract physiological information from living organisms. PPG takes advantage of complex light–matter interactions occurring when light at visible or near-infrared wavelengths impinges upon, and interacts with, human tissues or organs. The basic principle of PPG is that real-time changes of certain physiological parameters can affect physical processes governing the interaction of light with complex media (such as blood, skin, and bone), causing a measurable, modulated variation in the fraction of light that is either reflected and/or transmitted (Figure 2).

PPG has been used to study several biological tissues and organs, including the cardiovascular system, lungs, skin, muscles, and bones. Its primary use has been to assess blood flow-related phenomena. The features of PPG that contribute to its clinical utility are that it is non-invasive and that the pulse wave is synchronous with the cardiac cycle. Essentially, PPG can detect blood volume changes in the circulatory system of several organs including capillary beds and, therefore, its utility to date has been largely in circulatory diagnostics with a prime example being pulse oximetry.

Technological developments have revolutionized PPG devices, making them small and wearable while giving relatively accurate and robust information [20,21]. The devices can be worn on different parts of the body, where it is easy to measure the blood flow of an individual. From blood flow dynamics, the heart rate can be easily derived [22]. Some of the common types of heart rate monitors based on PPG include wrist wearables, chest bands, and smart rings. A limitation of PPG technology, which remains partially unaddressed and limits the reliability of the data, is the susceptibility of the signals to motion artifacts caused by movement. This problem may clearly affect wrist-worn heart rate measures during resistance training [22]. PPG-based heart rate measures should, therefore, be interpreted with caution, although they provide valuable information both during and after training.

Heart rate monitors are used during resistance training exercises to monitor the cardiovascular responses of the trainee. During intense physical activity, the body responds by increasing the heart rate to supply blood faster. Blood vessels expand to increase blood flow into the strained muscles. The heart rate monitors help to keep track of the rate at which the heart is pumping blood during resistance exercise workouts [22,23]. They are also used to monitor the intensity of the workouts in which a person is engaged. If an exercise is very intense in terms of difficulties and straining of muscles, the sensors indicate higher heart rates. The monitors can be used to help improve the quality of the workouts by ensuring that the trainee meets certain set goals. These insights improve intensity regulation and load management, and support individualized programming as well as progress tracking.

A heart rate monitor can be used to check the recovery progress between sets and workouts. It monitors heart rate both during rest and when exercising [24]. The heart rate monitor can check the speed at which the heart rate drops after engaging in a difficult strength exercise. When monitoring the heart rate during rest periods, it is relatively easy to determine whether the body has recovered sufficiently or not. Therefore, an informed decision can be made on whether and when to perform the next set of workouts. Another application of the devices is to track the overall fitness level in the long term [22]. Overall, heart rate monitors have the potential to help achieving fitness goals in resistance training in a safe and accurate manner. They ensure that there is no need to stop between workouts to check for heart rate. The data collected can be kept and shared with other devices and can be used for medical purposes if needed.

If combined with other sensors, such as temperature and movement sensors, PPGs also allow the extraction of information about sleep efficiency in an efficient manner. It should, however, be considered that estimates of sleep staging based on PPG are not always in line with those of reference polysomnography [25,26]. Considering that sleep may be affected by high-load training regimes [27], it is particularly helpful to track sleep-related indices of recovery after exercise. Offline analysis of heart rate during sleep can be performed to support the discrimination of light, deep, and rapid eye movement (REM) sleep phases. It is therefore possible to gain insights into the quality and duration of sleep and identify any patterns or trends over time. Overall, heart rate monitors are considered a useful tool for individuals looking to track and improve sleep quality and overall health [28].

### 3.2. Electrocardiography to Track Cardiac Output and Heart Rate Variability

An electrocardiogram (ECG) is a non-invasive and painless test that is carried out to measure the temporal evolution of the electrical activity of the heart, using electrodes placed on the chest. ECG signals are typically recorded by machines that consist of a set of electrodes connected to a central unit. Early ECG machines were constructed with analog electronics, where the signal drove a motor to print out the signal onto paper. Today, electrocardiographs use analog-to-digital converters to convert the electrical activity of the heart to a digital signal. Many ECG machines are now portable and commonly include a screen, a keyboard, and a printer on a wheeled cart. Recent advancements include the development of even smaller devices for inclusion in fitness trackers and smart watches. These smaller devices often rely on only two electrodes to deliver a single-lead ECG, however, porTable 12-lead devices powered by batteries are also available. Notably, these wireless ECG systems provide very similar performance to traditional ECG systems [29,30].

**Figure 3:**
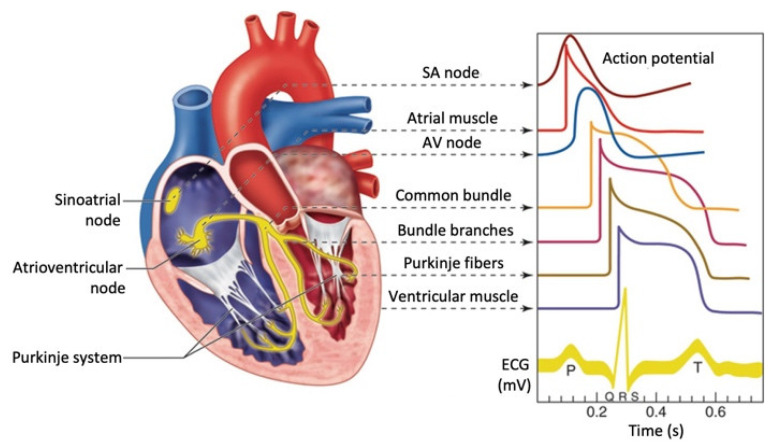
Cardiac electrical conduction system morphology and timing of action potentials from different regions of the heart. On the bottom right side, the ECG signal as measured on the body surface is shown.

The ECG electrodes detect the small changes in electrical potential that are a consequence of cardiac muscle depolarization followed by repolarization during each cardiac cycle (heartbeat). There are three main components to an ECG: the P wave, representing depolarization of the atria; the QRS complex, representing depolarization of the ventricles; and the T wave, representing repolarization of the ventricles (Figure 3) [31].

During each heartbeat, a healthy heart has an orderly progression of depolarization that starts with pacemaker cells in the sinoatrial node, spreads throughout the atrium, and passes through the atrioventricular node down into the bundle of His and into the Purkinje fibers, spreading down and to the left throughout the ventricles. This orderly pattern of depolarization gives rise to the characteristic ECG tracing. To the trained clinician, an ECG conveys a large amount of information about the structure of the heart and the function of its electrical conduction system [32]. Among other things, an ECG can be used to measure the rate and rhythm of heartbeats, the size and position of the heart chambers, the presence of any damage to the heart muscle cells or conduction system, the effects of drugs, and the function of implanted pacemakers. For a correct interpretation of the ECG signals, it is fundamental that a standard electrode positioning is performed. ECG recordings from misplaced electrodes can show distorted waveforms, increasing the likelihood of an incorrect diagnosis.

For adults, the use of ECG among those without symptoms or at low risk of cardiovascular disease is not indicated for prevention. This is because an ECG may falsely indicate the existence of a problem, leading to misdiagnosis, the recommendation of invasive procedures, and overtreatment. However, persons employed in certain critical occupations, such as aircraft pilots, may be required to have an ECG as part of their routine health evaluations. Hypertrophic cardiomyopathy screening may also be considered in individuals as part of sports physical screening out of concern for sudden cardiac failure. Overall, the ECG signal can be used as a vital parameter in the assessment of the overall health of the cardiovascular system [33]. If the person shows decreased cardiac output, this could be an indication of early signs of cardiac disease or other problems. Increased cardiac output could result from physiological changes associated with resistance training workouts. Therefore, assessing cardiac output before and during resistance training is important [34].

ECG signals can also be used to extract cardiac pulses with much higher temporal resolution than PPG. This allows the assessment of heart rate variability (HRV), which is the disparity in time intervals between successive heartbeats [35]. Several HRV parameters can be extracted, which may provide a better understanding of how the autonomic nervous system functions and the overall health status of the cardiovascular system [33]. Temporal HRV indices can provide measures of the probability of the person developing cardiovascular diseases. Further, spectral HRV indices can disentangle the specific responses of the sympathetic and parasympathetic nervous systems during exercise. Finally, nonlinear HRV methods have recently proven to be useful in aerobic and anaerobic threshold estimation [36].

### 3.3. Electromyography to Monitor Muscle Fatigue Conditions during Exercise

**Figure 4:**
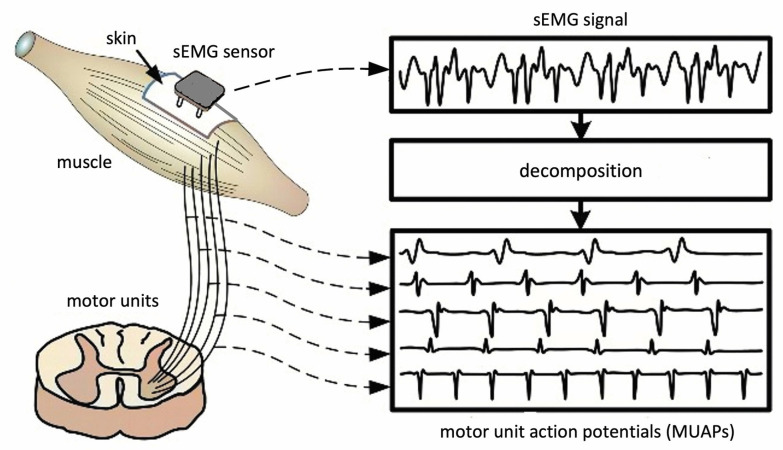
Acquisition of sMEG signal and decomposition into multiunit action potentials. Reprinted/adapted with permission from Ref. [37]. 2006, Journal of Neurophysiology.

When people perform physical exercises, their muscles go through a series of dynamic contractions. Surface electromyography (sEMG) sensors are used to measure the electrical activity produced within the muscles during contraction and can provide important data about the muscle activity levels at a given time [6]. sEMG signals are essentially comprised of superimposed motor unit action potentials (MUAPs) from several motor units (Figure 4) [37]. For a thorough analysis, the measured sEMG signals can be decomposed into their constituent MUAPs. MUAPs from different motor units tend to have different characteristic shapes, while those recorded by the same electrode from the same motor unit are typically similar. Notably, MUAP size and shape depend on where the electrode is located with respect to the fibers and, so, can appear to be different if the electrode changes position.

According to several studies, the changes in muscle fiber propagation velocity and the firing rate of muscle fibers could affect the sEMG power spectrum [6]. The power spectrum of the sEMG signal has been found to shift to a lower frequency during a sustained muscle contraction, and reductions of spectral parameters, such as the median frequency (MF) and the mean frequency (MNF), can be used as markers of muscle fatigue [38]. It should be noted that the sEMG signal easily combines different types of noise. Therefore, it is important that the background noise is removed from the sEMG signal to quantify muscle fatigue in a reliable manner.

For several years, sEMG signals have been collected using passive bipolar electrodes attached over the muscles and connected through wires to an acquisition device. An important advancement in EMG technology has been the introduction of wireless sEMG sensors [39]. These sensors are nowadays extensively validated and offer several advantages over traditional wired sEMG sensors [40,41,42]. First and foremost, wireless sEMG allows for greater freedom of movement during exercise, as there are no wires or cables connecting the muscle sensors to the recording device. This allows for more natural movement patterns and reduces the risk of interference or discomfort caused by the wires. Additionally, wireless sEMG is typically easier to set up and use, as there is no need to position and secure multiple wires onto the skin surface. This can save time and reduce the likelihood of errors or inaccuracies in the measurement process. For the reasons above, wireless sEMG is a valuable tool for researchers and practitioners looking to study muscle function and optimize resistance training programs.

### 3.4. Near-Infrared Spectroscopy to Measure Changes in Oxygen Saturation in Muscles

Muscle oxygenation sensors are highly beneficial because they are non-invasive, painless, and easy to apply. They entail the use of near-infrared spectroscopy (NIRS), a non-invasive technique that measures the concentration of oxygenated and deoxygenated hemoglobin in the blood [43]. NIRS shines a light on the body tissue, where the light penetrates the skin and muscle tissue to the hemoglobin molecules in the blood [43,44]. Some of the light is absorbed by the molecules and is measured, such that the level of oxygen saturation in the muscle tissue that was shone with the light is determined.

**Figure 5:**
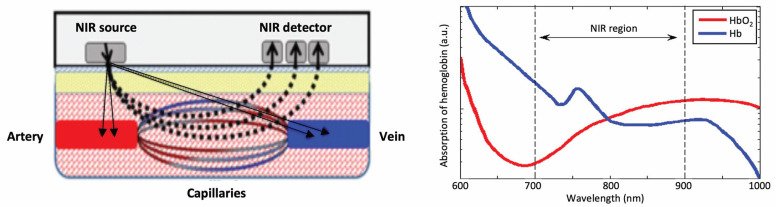
Schematic representation of NIRS signal propagation from the source to the detector, along with the spectra of absorption for Hb (blue) and HbO_2_ (red), respectively.

More specifically, the blood component hemoglobin scatters light, and the ratio of infrared light absorbed to those scattered changes depends on the degree of hemoglobin binding with oxygen. NIRS measures this rate of change and the change in oxygenated hemoglobin concentration. Near-infrared light having a wavelength of 700 to 900 nm is used in the optical measurement of living organisms. Visible light (wavelength 400 to 700 nm) is substantially absorbed by hemoglobin and other component organic matter, while absorption by water increases at wavelengths longer than near-infrared light. Thus, light in these wavelength ranges cannot internally penetrate living organisms. Though absorption of light in this wavelength region is caused mainly by oxygenated hemoglobin (HbO_2_) and deoxygenated hemoglobin (Hb), both of these have different absorption spectra, and the isosbestic point is in the vicinity of 805 nm (Figure 5) [45]. For this reason, if the molecular absorption coefficient of HbO_2_ and Hb is known, their concentration changes can be calculated by measuring the change in absorption at two or more wavelengths. Due to the physical principles on which it is built, an important limitation of the NIRS technology is that its light (wavelength 700 to 900 nm) penetrates only superficial layers.

When a person is engaged in resistance training, muscles require more oxygen due to the increased demand for energy [43]. The high demand can cause oxygen saturation in the muscle tissue to change. Muscle oxygenation sensors are placed on the skin in the region where the muscle under measurement is located. The sensors permit the reliable collection of data about muscle oxygenation in real time [46] and to determine the oxygen demand levels in the muscle during a resistance exercise [44]. There have been numerous studies involving muscle oxygenation sensors in the investigation of the effects of different training plans [43]. For instance, studies have detected variations in oxygen saturation within the quadriceps muscles when a person is doing single-leg press exercises, with an increase in oxygen saturation during the concentric phase and a decrease in the eccentric phase of the exercise [47].

### 3.5. Portable Metabolic Analyzers for Assessing Energy Expenditure and Metabolic Rate

Metabolic analyzers are devices that are used in the assessment of energy expenditure and metabolic rate. The analyzers measure the amount of oxygen consumed and carbon dioxide produced by the body [48]. Metabolic analyzers can be used in metabolic rate analysis of individuals engaged in physical exercise, helping in determining the energy disparities associated with different physical activities. They can also monitor the impact of the workouts and dietary interventions on metabolic rate and energy use [49].

Nearly every metabolic analyzer on the market uses a hose to directly connect the air exchanges from an athlete to the machine. A face mask covers the mouth and usually has a dual-shaped extender, with one valve that flows into the machine for readings. Because air contains moisture, filters exist to ensure the air is dry and clean for the readings, and to prevent health hazards to users later. Most machines need to be maintained and cleaned, but some have internal mechanisms that assist users and reduce maintenance requirements. Drift, a common problem with sensors, occurs when the measurement quality decays over time. To address this problem, a calibration is performed. Metabolic analyzers are usually calibrated using indirect calorimetry. The process entails the collection of expired air from the body and analyzing the oxygen and carbon dioxide content contained. Indirect calorimetry is used in the determination of the resting metabolic rate (RMR), which is the total amount of energy used by the body when resting to ensure that basic functions continue [49]. Some of the maintained body functions at rest include breathing, blood circulation, and digestion of food. In most people, about 60–75% of the daily energy use is accounted for by RMR. An alternative to indirect calorimetry, direct calorimetry devices can be used to directly measure heat production. They produce more accurate readings of metabolic rate compared to indirect calorimetry, but they are very expensive and must be operated by an expert [49]. Therefore, they are less common in both clinical and resistance workout settings.

Portable devices for metabolic analysis, which can be used outside a laboratory environment, have been recently introduced in the market [50]. These devices have similar performance as previously validated stationary metabolic systems [51,52]. Individuals following resistance training programs can use portable metabolic analyzers, as they are light and convenient to use [53]. Although these analyzers can be used in resistance training and other types of exercises, it should be considered that the metabolic data can only be interpreted by a person with specialized knowledge and expertise.

## 4. Wearable Devices for Measuring Biomechanical Parameters during Training

### 4.1. Inertial Measurement Units (IMUs) for Tracking Movement Patterns and Velocity

**Figure 6:**
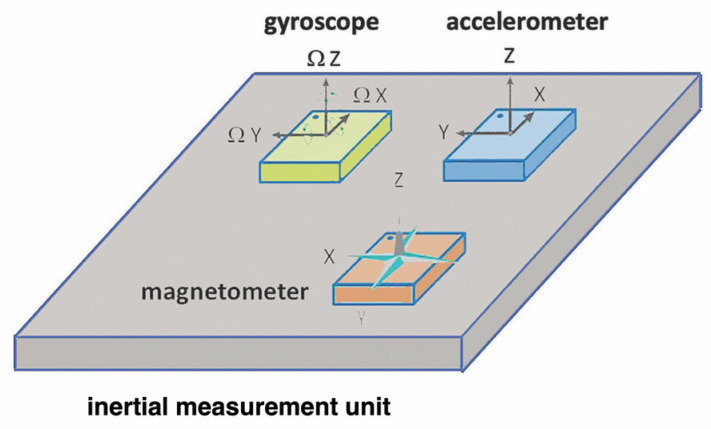
Schematic representation of an inertial measurement unit (IMU). The IMU is mounted on a platform consisting of sensors that provide different motion information: accelerometers, gyroscopes, and—optionally—magnetometers.

Inertial measurement units (IMUs) are comprised of small sensors fitted into wearable devices, which can be worn over different parts of the body. These wearable devices can be attached to the wrist, waist, or ankle, where they record and provide data about the orientation and movement of these body parts. The kinematic parameters extracted from IMUs provide excellent consistency with respect to gold-standard motion capture systems, both for upper [54] and lower limbs [55]. IMU sensors are composed of accelerometers, gyroscopes, and magnetometers (Figure 6), which measure linear acceleration, angular velocity, and magnetic field strength, respectively [56]. An accelerometer is the primary sensor responsible for measuring inertial acceleration, or the change in velocity over time, and can be found in a variety of different types, including mechanical accelerometers, quartz accelerometers, and micro-electromechanical system (MEMS) accelerometers. A MEMS accelerometer is essentially a mass suspended by a spring. The mass is known as the proof mass and the direction along which the mass is allowed to move is known as the sensitivity axis. When an accelerometer is subjected to a linear acceleration along the sensitivity axis, the acceleration causes the proof mass to shift to one side, with the amount of deflection proportional to the acceleration. A gyroscope is an inertial sensor which measures an object’s angular rate with respect to an inertial reference frame. There are many different types of gyroscopes available on the market, which range over various levels of performance and include mechanical gyroscopes, fiber-optic gyroscopes, ring laser gyroscopes, and quartz/MEMS gyroscopes. A magnetometer is a type of sensor that measures the strength and direction of a magnetic field. While there are many different types of magnetometers, most MEMS magnetometers rely on magneto-resistance to measure the surrounding magnetic field. Magneto-resistive magnetometers are comprised of permalloys that change resistance due to changes in magnetic fields. Typically, MEMS magnetometers are used to measure a local magnetic field which consists of a combination of Earth’s magnetic field as well as any magnetic fields created by nearby objects. The principal disadvantage of an IMU is that it is prone to errors that accumulate over time. The resulting drift is because the device is always measuring changes relative to itself, not triangulating against an absolute or known outside device.

Accelerometers embedded in IMUs can be used in determining the movement patterns and velocity in the resistance training period [24]. Importantly, the use of dedicated algorithms is required to assess the proper execution of the exercises and evaluate the quality of the motion [57,58,59]. A common application of accelerometers is the measure of the velocity at which the resistance equipment or tool is moved. For instance, coaches track this velocity to ensure that weights lifted are appropriate for the preferred effect and determine necessary adjustments to the total weights or number of repetitions appropriately. Accelerometers are also used to measure position over time, and, hence, motion range for a given movement. IMUs containing this kind of sensor are fitted on the body to track position and instant velocity [24], helping to determine whether movements were correctly executed during exercise. Gyroscopes can be used in resistance training, whereby measuring the rotation of the body will provide data on its orientation. This can be used to change and improve techniques and forms during resistance training. For instance, when a person is doing squats in an incorrect form, the gyroscope device will detect an incorrect body rotation and will then provide useful information on how to correct the movement. Data from both accelerometers and gyroscopes embedded in IMUs can be combined to give a more comprehensive insight into the movement of the body muscles during resistance training. They will offer bar speed, range motion, orientation, and feedback on technique and form.

### 4.2. Force Sensors for Measuring the Amount of Force Generated

Force sensors are used to measure the strength that is applied when engaged in an exercise. They generate crucial data for various groups, such as trainees, coaches, and researchers, to enable them to track fitness, optimize training sessions, and study biomechanics [60]. There are different types of force sensors that are used during exercises to collect data, including capacitive sensors, load cells, piezoelectric sensors, and strain gauge sensors [61]. Each of these sensors has benefits and problems depending on factors such as accuracy, durability, and sensitivity. Despite these issues, force sensors are generally considered the gold standard for force measurements.

The working mechanism of force sensors involves measuring the deformation of a material due to the application of a force. When a material is compressed or stretched there are changes in its electrical properties, allowing the sensors to determine the force that has been exerted. There are two types of force sensors, based on shunt mode and thru mode force resistors, respectively. Their functionality is identical, but they have different construction methods and properties. The former is more suitable for wide-pressure range sensing, whereas the latter is for light-pressure precise sensing. These devices can be integrated into different types of exercise tools and equipment, including treadmills and rowing machines [61]. Some of the sensors have been modified to fit on different body parts as wearables, where they collect information and provide feedback about the exercise. For instance, they can be fitted on an athlete to measure forces generated during exercises such as running, jumping, and pushups [60]. For accuracy and consistency in measurements, force sensors must be calibrated appropriately. They must give accurate data to ensure that the evaluation of the exercise performance and progress gives results that will promote performance improvement.

Force sensors have limitations that hinder them from becoming reliable in resistance training. The data generated require some level of expertise for a person to understand and use them to make appropriate changes. Also, these sensors may produce incorrect information when force is applied at an angle. The force sensors are also affected by environmental factors such as temperature and humidity, and, thus, they are likely to provide incorrect information [61]. Furthermore, it is challenging to determine which force sensor is appropriate for a particular exercise. A combination of several force sensors could help in increasing the accuracy of the information generated during an exercise.

### 4.3. Pressure Sensors for Assessing Foot Pressure and Balance

Insole pressure sensors can be used to measure foot pressure and balance. They are designed in a manner to quantify how pressure is distributed on the foot when a person is engaging in various resistance exercises and movements [62]. Insole pressure sensors are placed inside the shoe that is used in the training exercises. They are very thin and flexible to ensure that they do not cause any discomfort to the person wearing the shoe. On the other hand, their performance is limited by the sole’s thickness, external influences such as water, humidity, sweat, and changes in temperature, which have been considered using compensation techniques to guarantee accurate measurements. Validation studies show that insole pressure sensors have a high measurement reproducibility, although they may underestimate the vertical ground reaction force [63,64].

Insole pressure sensors are used in resistance training, where they can help in identifying imbalances and asymmetries occurring in foot pressure and balance when a person is doing exercises. Sometimes, a person may exert more pressure on one foot when squatting or weightlifting. This can easily cause uneven muscle development in the legs [65]. With information from the pressure sensors, the trainee can use it to improve the technique and other changes to ensure that there is equal distribution of pressure on both legs and minimal risks of injuries to the muscles [60]. The pressure sensors can be used to track foot pressure changes over a period, and this will help in evaluating the progress achieved. The information can be used to make changes to the resistance training program so that there can be better performance in the long term [66].

## 5. Applications of Wearables in Resistance Training Research

### 5.1. Assessment of Training Load and Fatigue

In resistance training, an important variable is the training load. Adjusting the load helps ensure that the risks of injury are minimized, there is an adequate adaptation to the training, and the recovery process is sufficient [60]. The changes are made to increase or decrease fatigue. As extremely intense physical activities in resistance training could induce severe muscle injuries, a trainer could monitor physiological and biomechanical parameters to decide whether the training should be amended. The best wearables to use in tracking training load are heart rate monitors, muscle activity sensors, and IMUs.

Heart rate monitoring is more commonly associated with cardiovascular exercises like running or cycling and is rarely applied during strength-focused sports such as powerlifting. However, heart rate monitoring during resistance training generally renders valuable information about exertion level and helps tailor workouts for better outcomes [67]. Firstly, it assists with predicting exercise intensity, allowing individuals to work within a desired target heart rate zone. This helps to optimize the training stimulus and adjust the load or number of repetitions accordingly. Secondly, it provides information on the cardiovascular response, highlighting the stress imposed on the cardiovascular system during the training session. Furthermore, heart rate monitoring during rest intervals allows for a more accurate assessment of recovery capacity, ensuring appropriate recovery intervals are implemented between sets to maintain optimal performance and prevent overexertion.

Another important variable to consider is muscle fatigue due to its impact on exercise performance, risks of injuries, and muscle activation patterns [68]. Wearable devices such as sEMG sensors can be used to measure the electrical activities of different muscles when exercising in real time [69,70]. Therefore, it is relatively easy to identify the point at which the muscle begins to fatigue during a training session. The data can be used to optimize the training protocol to attenuate or eliminate fatigue. The number of sets can be decreased or slower to give the muscles time to relax. Other devices, such as muscle oxygen saturation sensors, are also important in assessing muscle fatigue [43], as they show the ability of muscle tissues to produce energy through aerobic processes. In addition, IMUs can be used in the monitoring of fatigue during resistance workout exercises and keep track of the changes occurring in the joint angles and velocity over time [71,72]. They provide further information on movement patterns that are causing too much fatigue when the training load is standardized. For instance, when a person begins to show signs of fatigue between deadlifts, IMUs are expected to show a reduced lift speed. It is hereby possible to track the effects of fatigue on the biomechanics of the body and make appropriate adjustments to the workouts [73].

### 5.2. Optimization of Exercise Technique and Performance

Resistance training aims at giving muscles more strength, power, and endurance. Therefore, it is critical to ensure that the exercise techniques are optimized for better performance. One of the key factors to consider is the application of proper form and technique when training. Each exercise has specific techniques and forms used to enhance the safety of the trainee and produce maximum results [74]. Learning the correct form and technique should be prioritized and practiced consistently to alleviate injuries and improve performance [75]. A good example, in this case, is the technique and form of performing squats. IMUs and the insights they provide regarding joint angles and movement patterns render valuable biomechanical information on how to augment one’s technique to perform a certain skill or movement more efficiently and correctly. To perform a technically correct squat, for example, the trainee should initiate the movement by hinging at the hips, keeping the chest up and back straight, lowering the body by bending the knees while keeping them in line with the toes, and driving through the heels to return to the starting position. As there are many degrees of freedom during this exercise, IMUs drastically facilitate measuring (in)correct movements so they can be adequately adjusted to optimize training and lower the risk of becoming injured.

### 5.3. Monitoring of Recovery and Injury Prevention

Importantly, training adaptations primarily occur during the recovery period after a fatiguing exercise. Hence, the subtle balance between intensity and recovery must be adequately maintained to avoid overtraining and reduce the risk of injuries [74]. The recovery period comprises resting to give muscles the time to heal from the tearing caused by the resistance exercises and let them grow. Recovery can be monitored by tracking sleep quality and quantity, checking HRV, and monitoring energy levels and fatigue [6,76,77,78,79].

To optimize recovery, it should be noted that nutrition is integral to muscle growth. As such, nutritious and balanced diets should be taken, and staying hydrated should be promoted when exercising. The type of diet which an individual enrolled in a resistance training program follows significantly influences performance [80]. For example, consuming whey protein after strength training helps promote muscle recovery and growth by providing the necessary amino acids for protein synthesis. The effects of such dietary interventions on body metabolism can be efficiently monitored using portable metabolic analysers.

Some of the injuries can be severe to the extent of ending ambitions in resistance workouts. Therefore, injury prevention is also important in the optimization of exercise technique and performance. To this end, it can be beneficial to monitor information on muscle oxygenation and fatigue, respectively, extracted from NIRS and sEMG signals [81]. Indeed, stiff muscles which are subjected to sudden intense exercises can easily be torn, leading to a serious injury [74]. Additionally, many people engage in resistance exercises without giving their bodies sufficient time to rest and recover. Adequate recovery is crucial to allow muscles to heal. In addition, sleep as the cornerstone of recovery, should be prioritized to enable muscles to optimally rest, grow, and recover [74]. Moreover, overtraining can lead to poor outcomes due to injuries and muscle damage that were not given enough time to heal and recover. Therefore, wearable technology can assist in monitoring whether sufficient rest between workouts is obtained to sustainably improve performance.

## 6. Limitations of Wearable Technology in Resistance Training Research

### 6.1. Accuracy and Reliability of Measurements

There are several factors on which the accuracy and reliability of measurements from wearable devices depend. These multiple factors form one of the biggest challenges that wearable technology faces in resistance training research. The first determiner is the type of sensor technology used in the device. Some wearable devices have more advanced sensors that detect and collect more accurate data compared to others. However, most of these advanced sensors are expensive, and only a few people can afford them during resistance training research. Of course, the cost of the wearable devices must be put in balance with the quality of the signals that are collected.

Another major determiner is the location and placement of the sensor on the body part. The sensor must be placed in an area where it will detect and collect the most information from the body for it to be accurate and reliable. For instance, a wireless sEMG sensor is often used to measure muscle activation when a person is engaged in resistance training workouts. The position of the sEMG sensor significantly affects the quality and reliability of the information collected. When the device is placed further from the muscle that is to be activated, its sensitivity is reduced, and, thus, it will not provide accurate data [73]. Another example is the accelerometer sensor that tracks the movements of the body when exercising. The accuracy and reliability of the accelerometer sensor can be affected negatively by factors such as the orientation of the device, placement of the sensor, and the type of exercise during resistance training [24].

Researchers can address some of these challenges that hinder the accuracy and reliability of measurements from wearable devices through extensive pilot and validation studies. They will have to test various sensors and placements, calibrate devices for different exercises, develop and adjust data processing algorithms, and use different individuals for a particular exercise. This course of action will help in increasing the accuracy and reliability of wearable sensor technologies to produce better measurements during resistance training.

### 6.2. Validation and Standardization of Wearable Technology

Wearable technology is also faced with the challenge of limited validation and standardization in resistance training research. Most of the wearable devices used in resistance training are not thoroughly validated or standardized. This can cause inconsistencies in the data that the devices collect. Therefore, it is challenging to compare results across different studies and develop a conclusive protocol [74]. Validation is a process that involves the assessment of the accuracy and reliability of a measurement tool, such as a wearable technology, against proven standards. When conducting resistance training studies, validation will entail the comparison of measurements collected from a sensor device to those obtained through the gold standard laboratory equipment. On the other hand, standardization involves the establishment of consistent protocols and procedures that are to be used in measurement tools in different research environments and studies. Some of the protocols for wearable devices may include data collection and analysis procedures, calibration, and device attachment.

There are many implications of having limited validation and standardization of wearable technology used in resistance training research. For instance, this can lead to inconsistencies in collected data using the devices. The inconsistencies make it difficult to produce a conclusive result on different factors of resistance training that affect performance and other outcomes. Another implication is the difficulty in comparing the results of different studies, which hinders the development of evidence-based recommendations for resistance training. In this respect, researchers should be encouraged to collaborate with manufacturers and other experts in this field to define validation mechanisms and standardized protocols for resistance training [1].

### 6.3. Ethical Considerations and Privacy Concerns

The popularity of wearable devices in resistance training has increased significantly in recent years. It provides users with real-time data about various parts and systems of the body. Despite having numerous benefits, there are several ethical considerations and privacy concerns that must be addressed. One ethical concern relates to the accuracy and reliability of wearable devices [7,82]. Users should be informed about the limitations and possible errors in the collected data from each wearable device being used. It should be clear that getting unrealistic results might lead them to make wrong decisions on their training plans and to be disappointed with the results obtained.

Another concern is on the matter of informed consent [83]. It is important to ensure that users of these devices are fully informed about the purpose and functionality of the devices before fitting them onto their bodies. Also, it should be explained to them about the personal data that will be collected, its use, and its being shared with others. Every person under resistance training has the right to be informed before agreeing to use wearable devices [83]. Each person who is uncomfortable with the technology should have the right to refuse consent.

Additionally, there are risks of cybersecurity and data breaches when sharing personal information. Wearable devices are typically capable of sharing the sensitive data collected with other devices, such as computers and smartphones [7]. The sensitive data may include personal details, health status, and exercise habits. Caution is needed when sharing this data between devices to prevent instances of data breaches by unauthorized people. To address the ethical implications of cybersecurity and data breaches, the user is supposed to maintain anonymity and also have control of their personal information [83]. Individuals undergoing resistance training, as well as their coaches, should ensure that neither the person being trained nor the coach expose any data to anyone else not involved in the program.

## 7. Conclusions and Future Perspectives

The use of wearables has the potential to revolutionize resistance training research by providing accurate and objective data on various physiological and biomechanical parameters during training. Wearables technology has been rapidly evolving over the years, and there are several trends emerging in the field. It is worth mentioning, for instance, that wearable devices are becoming available to track brain activity [84,85]. This may open new areas of research aimed at examining neuroplasticity effects induced by resistance training [86] and at monitoring central fatigue during exercise [87]. Smart clothing is another emerging trend in wearable technology, with sensors and other components integrated into items such as shirts, pants, and jackets. Their general use is expected to rapidly increase, providing accurate information on body kinematics in a wide range of training conditions [88].

Overall, the combined use of different wearables is warranted to provide crucial information on the mutual relationships between physiological and biomechanical parameters measured during training programs [6,7]. Despite the potential benefits related to the use of wearables in resistance training research, there are also important challenges and limitations that will need to be addressed. Further research is needed to establish the reliability and validity of wearable devices and to integrate them into standardized resistance training protocols. Importantly, the issue of cybersecurity should also be considered when gathering large amounts of health data through wearables [7]. To address this problem, robust data encryption and protection techniques should be implemented in wearable devices.

The creation of repositories of wearable data for resistance training research can certainly be an exciting avenue, as this would open the way for the use of artificial intelligence (AI) techniques [89,90]. For an effective use of AI, these repositories should contain a large amount of high-quality data sets. A potential application is the prediction of the outcomes of resistance training programs based on various parameters such as age, sex, fitness level, training history, and nutrition. AI can also be used for virtual coaching, by analysing biophysical parameters in real time and providing suggestions on how to optimize the resistance training program [91]. This information can be provided to the individual by a voice-based virtual agent, but also through the use of augmented reality or virtual reality technology [92].

In summary, we posit that AI applied to wearable data has the potential to revolutionize resistance training research by providing new insights, improving the effectiveness of training programs, and enhancing the overall experience for individuals during exercise.

## References

[1] Carvalho L., Junior R.M., Barreira J., Schoenfeld B.J., Orazem J., Barroso R. (2022). Muscle Hypertrophy and Strength Gains after Resistance Training with Different Volume-Matched Loads: A Systematic Review and Meta-Analysis. Appl. Physiol. Nutr. Metab..

[2] Westcott W.L. (2012). Resistance Training Is Medicine: Effects of Strength Training on Health. Curr. Sports Med. Rep..

[3] Cruz-Jentoft A.J., Sayer A.A. (2019). Sarcopenia. Lancet.

[4] Yasuda T. (2022). Selected Methods of Resistance Training for Prevention and Treatment of Sarcopenia. Cells.

[5] Schoenfeld B.J., Grgic J., Ogborn D., Krieger J.W. (2017). Strength and Hypertrophy Adaptations Between Low- vs. High-Load Resistance Training: A Systematic Review and Meta-Analysis. J. Strength Cond. Res..

[6] Sperlich B., Aminian K., Düking P., Holmberg H.-C. (2019). Editorial: Wearable Sensor Technology for Monitoring Training Load and Health in the Athletic Population. Front. Physiol..

[7] Dunn J., Runge R., Snyder M. (2018). Wearables and the Medical Revolution. Per. Med..

[8] Mcleod J.C., Stokes T., Phillips S.M. (2019). Resistance Exercise Training as a Primary Countermeasure to Age-Related Chronic Disease. Front. Physiol..

[9] Martins A.D., Fernandes O., Pereira A., Oliveira R., Alderete Goñi F.D., Leite N.J.C., Brito J.P. (2022). The Effects of High-Speed Resistance Training on Health Outcomes in Independent Older Adults: A Systematic Review and Meta-Analysis. Int. J. Environ. Res. Public Health.

[10] Khodadad Kashi S., Mirzazadeh Z.S., Saatchian V. (2023). A Systematic Review and Meta-Analysis of Resistance Training on Quality of Life, Depression, Muscle Strength, and Functional Exercise Capacity in Older Adults Aged 60 Years or More. Biol. Res. Nurs..

[11] Currier B.S., Mcleod J.C., Banfield L., Beyene J., Welton N.J., D’Souza A.C., Keogh J.A.J., Lin L., Coletta G., Yang A. (2023). Resistance Training Prescription for Muscle Strength and Hypertrophy in Healthy Adults: A Systematic Review and Bayesian Network Meta-Analysis. Br. J. Sports Med..

[12] Schoenfeld B.J., Ratamess N.A., Peterson M.D., Contreras B., Tiryaki-Sonmez G. (2015). Influence of Resistance Training Frequency on Muscular Adaptations in Well-Trained Men. J. Strength Cond. Res..

[13] Morrissey M.C., Harman E.A., Johnson M.J. (1995). Resistance Training Modes: Specificity and Effectiveness. Med. Sci. Sports Exerc..

[14] Kraemer W.J., Ratamess N.A. (2004). Fundamentals of Resistance Training: Progression and Exercise Prescription. Med. Sci. Sports Exerc..

[15] Skorski S., Mujika I., Bosquet L., Meeusen R., Coutts A.J., Meyer T. (2019). The Temporal Relationship Between Exercise, Recovery Processes, and Changes in Performance. Int. J. Sports Physiol. Perform..

[16] Kellmann M., Bertollo M., Bosquet L., Brink M., Coutts A.J., Duffield R., Erlacher D., Halson S.L., Hecksteden A., Heidari J. (2018). Recovery and Performance in Sport: Consensus Statement. Int. J. Sports Physiol. Perform..

[17] Torous J., Stern A.D., Bourgeois F.T. (2022). Regulatory Considerations to Keep Pace with Innovation in Digital Health Products. NPJ Digit. Med..

[18] Awad S., Aljuburi L., Lumsden R.S., Mpandzou M., Marinus R. (2023). Connected Health in US, EU, and China: Opportunities to Accelerate Regulation of Connected Health Technologies to Optimize Their Role in Medicines Development. Front. Med..

[19] Baskan A., Goncu-Berk G. (2022). User Experience of Wearable Technologies: A Comparative Analysis of Textile-Based and Accessory-Based Wearable Products. Appl. Sci..

[20] Gupta K., Sinhal R., Badhiye S.S. (2023). Remote Photoplethysmography-Based Human Vital Sign Prediction Using Cyclical Algorithm. J. Biophotonics.

[21] Correia B., Dias N., Costa P., Pêgo J.M. (2020). Validation of a Wireless Bluetooth Photoplethysmography Sensor Used on the Earlobe for Monitoring Heart Rate Variability Features during a Stress-Inducing Mental Task in Healthy Individuals. Sensors.

[22] Zhang Y., Weaver R.G., Armstrong B., Burkart S., Zhang S., Beets M.W. (2020). Validity of Wrist-Worn Photoplethysmography Devices to Measure Heart Rate: A Systematic Review and Meta-Analysis. J. Sports Sci..

[23] Charlton P.H., Kyriacou P., Mant J., Alastruey J. (2020). Acquiring Wearable Photoplethysmography Data in Daily Life: The PPG Diary Pilot Study. Eng. Proc..

[24] Kinnunen H., Häkkinen K., Schumann M., Karavirta L., Westerterp K.R., Kyröläinen H. (2019). Training-Induced Changes in Daily Energy Expenditure: Methodological Evaluation Using Wrist-Worn Accelerometer, Heart Rate Monitor, and Doubly Labeled Water Technique. PLoS ONE.

[25] Wulterkens B.M., Fonseca P., Hermans L.W.A., Ross M., Cerny A., Anderer P., Long X., van Dijk J.P., Vandenbussche N., Pillen S. (2021). It Is All in the Wrist: Wearable Sleep Staging in a Clinical Population versus Reference Polysomnography. Nat. Sci. Sleep.

[26] Liu P.-K., Ting N., Chiu H.-C., Lin Y.-C., Liu Y.-T., Ku B.-W., Lee P.-L. (2023). Validation of Photoplethysmography and Acceleration-Based Sleep Staging in a Community Sample: Comparison with Polysomnography and Actiwatch. J. Clin. Sleep Med..

[27] Chennaoui M., Arnal P.J., Sauvet F., Léger D. (2015). Sleep and Exercise: A Reciprocal Issue?. Sleep Med. Rev..

[28] Stein P.K., Pu Y. (2012). Heart Rate Variability, Sleep and Sleep Disorders. Sleep Med. Rev..

[29] Wang J.-J., Liu S.-H., Tsai C.-H., Manousakas I., Zhu X., Lee T.-L. (2023). Signal Quality Analysis of Single-Arm Electrocardiography. Sensors.

[30] Lee M.H., Jang G.Y., Kim Y.E., Yoo P.J., Wi H., Oh T.I., Woo E.J. (2018). Portable Multi-Parameter Electrical Impedance Tomography for Sleep Apnea and Hypoventilation Monitoring: Feasibility Study. Physiol. Meas..

[31] Dobrzynski H., Anderson R.H., Atkinson A., Borbas Z., D’Souza A., Fraser J.F., Inada S., Logantha S.J.R.J., Monfredi O., Morris G.M. (2013). Structure, Function and Clinical Relevance of the Cardiac Conduction System, Including the Atrioventricular Ring and Outflow Tract Tissues. Pharmacol. Ther..

[32] Bayram M., Yancy C.W. (2009). Transthoracic Impedance Cardiography: A Noninvasive Method of Hemodynamic Assessment. Heart Fail. Clin..

[33] Sharma S., Merghani A., Mont L. (2015). Exercise and the Heart: The Good, the Bad, and the Ugly. Eur. Heart J..

[34] Pingitore A., Peruzzi M., Clarich S.C., Palamà Z., Sciarra L., Cavarretta E. (2023). An Overview of the Electrocardiographic Monitoring Devices in Sports Cardiology: Between Present and Future. Clin. Cardiol..

[35] Xhyheri B., Manfrini O., Mazzolini M., Pizzi C., Bugiardini R. (2012). Heart Rate Variability Today. Prog. Cardiovasc. Dis..

[36] Rogers B., Gronwald T. (2022). Fractal Correlation Properties of Heart Rate Variability as a Biomarker for Intensity Distribution and Training Prescription in Endurance Exercise: An Update. Front. Physiol..

[37] De Luca C.J., Adam A., Wotiz R., Gilmore L.D., Nawab S.H. (2006). Decomposition of Surface EMG Signals. J. Neurophysiol..

[38] Bartuzi P., Roman-Liu D. (2014). Assessment of Muscle Load and Fatigue with the Usage of Frequency and Time-Frequency Analysis of the EMG Signal. Acta Bioeng. Biomech..

[39] Budinger T.F. (2003). Biomonitoring with Wireless Communications. Annu. Rev. Biomed. Eng..

[40] Örücü S., Selek M. (2019). Design and Validation of Multichannel Wireless Wearable SEMG System for Real-Time Training Performance Monitoring. J. Healthc. Eng..

[41] Liang Z., Wang X., Guo J., Ye Y., Zhang H., Xie L., Tao K., Zeng W., Yin E., Ji B. (2023). A Wireless, High-Quality, Soft and Portable Wrist-Worn System for SEMG Signal Detection. Micromachines.

[42] Gomez-Correa M., Cruz-Ortiz D. (2022). Low-Cost Wearable Band Sensors of Surface Electromyography for Detecting Hand Movements. Sensors.

[43] Klusiewicz A., Rębiś K., Ozimek M., Czaplicki A. (2021). The Use of Muscle Near-Infrared Spectroscopy (NIRS) to Assess the Aerobic Training Loads of World-Class Rowers. Biol. Sport.

[44] Paulauskas R., Nekriošius R., Dadelienė R., Sousa A., Figueira B. (2022). Muscle Oxygenation Measured with Near-Infrared Spectroscopy Following Different Intermittent Training Protocols in a World-Class Kayaker—A Case Study. Sensors.

[45] Sanni A.A., McCully K.K. (2019). Interpretation of Near-Infrared Spectroscopy (NIRS) Signals in Skeletal Muscle. J. Funct. Morphol. Kinesiol..

[46] Benni P.B., MacLeod D., Ikeda K., Lin H.-M. (2018). A Validation Method for Near-Infrared Spectroscopy Based Tissue Oximeters for Cerebral and Somatic Tissue Oxygen Saturation Measurements. J. Clin. Monit. Comput..

[47] Miranda-Fuentes C., Chirosa-Ríos L.J., Guisado-Requena I.M., Delgado-Floody P., Jerez-Mayorga D. (2021). Changes in Muscle Oxygen Saturation Measured Using Wireless Near-Infrared Spectroscopy in Resistance Training: A Systematic Review. Int. J. Environ. Res. Public Health.

[48] Hodges L.D., Brodie D.A., Bromley P.D. (2005). Validity and Reliability of Selected Commercially Available Metabolic Analyzer Systems. Scand. J. Med. Sci. Sports.

[49] Sordi A.F., Mariano I.R., Silva B.F., Magnani Branco B.H. (2022). Resting Metabolic Rate in Bodybuilding: Differences between Indirect Calorimetry and Predictive Equations. Clin. Nutr. ESPEN.

[50] Robles-González L., Gutiérrez-Hellín J., Aguilar-Navarro M., Ruiz-Moreno C., Muñoz A., Del-Coso J., R Ruiz J., Amaro-Gahete F.J. (2021). Inter-Day Reliability of Resting Metabolic Rate and Maximal Fat Oxidation during Exercise in Healthy Men Using the Ergostik Gas Analyzer. Nutrients.

[51] Tsekouras Y.E., Tambalis K.D., Sarras S.E., Antoniou A.K., Kokkinos P., Sidossis L.S. (2019). Validity and Reliability of the New Portable Metabolic Analyzer PNOE. Front. Sports Act. Living.

[52] Yao P., Guo W., Sheng X., Zhang D., Zhu X. (2014). A Portable Multi-Channel Wireless NIRS Device for Muscle Activity Real-Time Monitoring. Annu. Int. Conf. IEEE Eng. Med. Biol. Soc..

[53] Seo H.-C., Shin D., Leem C.H., Joo S. (2021). Development of a Portable Respiratory Gas Analyzer for Measuring Indirect Resting Energy Expenditure (REE). J. Healthc. Eng..

[54] Lee R., Akhundov R., James C., Edwards S., Snodgrass S.J. (2023). Variations in Concurrent Validity of Two Independent Inertial Measurement Units Compared to Gold Standard for Upper Body Posture during Computerised Device Use. Sensors.

[55] Zhang H., Song Y., Li C., Dou Y., Wang D., Wu Y., Chen X., Liu D. (2023). Validation of a Wearable System for Lower Extremity Assessment. Orthop. Surg..

[56] Samatas G.G., Pachidis T.P. (2022). Inertial Measurement Units (IMUs) in Mobile Robots over the Last Five Years: A Review. Designs.

[57] Sabatini A.M. (2011). Estimating Three-Dimensional Orientation of Human Body Parts by Inertial/Magnetic Sensing. Sensors.

[58] Yi C., Ma J., Guo H., Han J., Gao H., Jiang F., Yang C. (2018). Estimating Three-Dimensional Body Orientation Based on an Improved Complementary Filter for Human Motion Tracking. Sensors.

[59] Hoang T., Shiao Y. (2023). New Method for Reduced-Number IMU Estimation in Observing Human Joint Motion. Sensors.

[60] Hernández A.L., Barrera Cortés M.C., Barón A.Á., TéllezTinjacá L.A., Guío Á.H.A. (2022). Competitive Advantage of Wearable Technology in Sports Training. Wearable Technol..

[61] Sander J., Kumme R. (2021). Comparison of Force Measuring Devices with Static and Continuous Loading. Meas. Sens..

[62] Jiang Y., Wang D., Ying J., Chu P., Qian Y., Chen W. (2021). Design and Preliminary Validation of Individual Customized Insole for Adults with Flexible Flatfeet Based on the Plantar Pressure Redistribution. Sensors.

[63] Lambrich J., Hagen M., Schwiertz G., Muehlbauer T. (2023). Concurrent Validity and Test-Retest Reliability of Pressure-Detecting Insoles for Static and Dynamic Movements in Healthy Young Adults. Sensors.

[64] Burch K., Doshi S., Chaudhari A., Thostenson E., Higginson J. (2023). Estimating Ground Reaction Force with Novel Carbon Nanotube-Based Textile Insole Pressure Sensors. Wearable Technol..

[65] Rivera C.E. (2016). Core and Lumbopelvic Stabilization in Runners. Phys. Med. Rehabil. Clin. N. Am..

[66] Morin P., Muller A., Pontonnier C., Dumont G. (2022). Evaluation of the Foot Center of Pressure Estimation from Pressure Insoles during Sidestep Cuts, Runs and Walks. Sensors.

[67] Ludwig M., Hoffmann K., Endler S., Asteroth A., Wiemeyer J. (2018). Measurement, Prediction, and Control of Individual Heart Rate Responses to Exercise-Basics and Options for Wearable Devices. Front. Physiol..

[68] Elshafei M., Shihab E. (2021). Towards Detecting Biceps Muscle Fatigue in Gym Activity Using Wearables. Sensors.

[69] Sorbie G.G., Williams M.J., Boyle D.W., Gray A., Brouner J., Gibson N., Baker J.S., Easton C., Ugbolue U.C. (2018). Intra-Session and Inter-Day Reliability of the Myon 320 Electromyography System During Sub-Maximal Contractions. Front. Physiol..

[70] Belbasis A., Fuss F.K. (2018). Muscle Performance Investigated with a Novel Smart Compression Garment Based on Pressure Sensor Force Myography and Its Validation Against EMG. Front. Physiol..

[71] Clemente F.M., Akyildiz Z., Pino-Ortega J., Rico-González M. (2021). Validity and Reliability of the Inertial Measurement Unit for Barbell Velocity Assessments: A Systematic Review. Sensors.

[72] Chambers R., Gabbett T.J., Cole M.H., Beard A. (2015). The Use of Wearable Microsensors to Quantify Sport-Specific Movements. Sports Med..

[73] Chen L., Liu X., Xuan B., Zhang J., Liu Z., Zhang Y. (2021). Selection of EMG Sensors Based on Motion Coordinated Analysis. Sensors.

[74] Krzysztofik M., Wilk M., Wojdała G., Gołaś A. (2019). Maximizing Muscle Hypertrophy: A Systematic Review of Advanced Resistance Training Techniques and Methods. Int. J. Environ. Res. Public Health.

[75] Adesida Y., Papi E., McGregor A.H. (2019). Exploring the Role of Wearable Technology in Sport Kinematics and Kinetics: A Systematic Review. Sensors.

[76] Cao R., Azimi I., Sarhaddi F., Niela-Vilen H., Axelin A., Liljeberg P., Rahmani A.M. (2022). Accuracy Assessment of Oura Ring Nocturnal Heart Rate and Heart Rate Variability in Comparison with Electrocardiography in Time and Frequency Domains: Comprehensive Analysis. J. Med. Internet Res..

[77] Altini M., Kinnunen H. (2021). The Promise of Sleep: A Multi-Sensor Approach for Accurate Sleep Stage Detection Using the Oura Ring. Sensors.

[78] Roberts D.M., Schade M.M., Mathew G.M., Gartenberg D., Buxton O.M. (2020). Detecting Sleep Using Heart Rate and Motion Data from Multisensor Consumer-Grade Wearables, Relative to Wrist Actigraphy and Polysomnography. Sleep.

[79] Ko P.-R.T., Kientz J.A., Choe E.K., Kay M., Landis C.A., Watson N.F. (2015). Consumer Sleep Technologies: A Review of the Landscape. J. Clin. Sleep Med..

[80] Henselmans M., Bjørnsen T., Hedderman R., Vårvik F.T. (2022). The Effect of Carbohydrate Intake on Strength and Resistance Training Performance: A Systematic Review. Nutrients.

[81] Di Giminiani R., Cardinale M., Ferrari M., Quaresima V. (2020). Validation of Fabric-Based Thigh-Wearable EMG Sensors and Oximetry for Monitoring Quadriceps Activity during Strength and Endurance Exercises. Sensors.

[82] Li R.T., Kling S.R., Salata M.J., Cupp S.A., Sheehan J., Voos J.E. (2016). Wearable Performance Devices in Sports Medicine. Sports Health.

[83] Tu J., Gao W. (2021). Ethical Considerations of Wearable Technologies in Human Research. Adv. Healthc. Mater..

[84] Krigolson O.E., Hammerstrom M.R., Abimbola W., Trska R., Wright B.W., Hecker K.G., Binsted G. (2021). Using Muse: Rapid Mobile Assessment of Brain Performance. Front. Neurosci..

[85] Mascia A., Collu R., Spanu A., Fraschini M., Barbaro M., Cosseddu P. (2023). Wearable System Based on Ultra-Thin Parylene C Tattoo Electrodes for EEG Recording. Sensors.

[86] Hortobágyi T., Granacher U., Fernandez-Del-Olmo M., Howatson G., Manca A., Deriu F., Taube W., Gruber M., Márquez G., Lundbye-Jensen J. (2021). Functional Relevance of Resistance Training-Induced Neuroplasticity in Health and Disease. Neurosci. Biobehav. Rev..

[87] Gorzi A., Rezapour N., Jabbari S., Youzbashi L., Salehi J., Gahreman D., Krause Neto W. (2022). Deceptive Intensities: An Exploratory Strategy for Overcoming Early Central Fatigue in Resistance Training. Physiol. Behav..

[88] Bryson D. (2023). Smart Clothing and Wearable Technology in Medical and Healthcare Applications. Smart Clothes Wearable Technol..

[89] Nahavandi D., Alizadehsani R., Khosravi A., Acharya U.R. (2022). Application of Artificial Intelligence in Wearable Devices: Opportunities and Challenges. Comput. Methods Programs Biomed..

[90] Yu K.-H., Beam A.L., Kohane I.S. (2018). Artificial Intelligence in Healthcare. Nat. Biomed. Eng..

[91] Lydakis A., Meng Y., Munroe C., Wu Y.-N., Begum M. (2017). A Learning-Based Agent for Home Neurorehabilitation. IEEE Int. Conf. Rehabil. Robot..

[92] Le Noury P., Polman R., Maloney M., Gorman A. (2022). A Narrative Review of the Current State of Extended Reality Technology and How It Can Be Utilised in Sport. Sports Med..

